# Beyond BMI: The Role of Visceral Adipose Tissue in Cardiometabolic Risk Stratification Among Children and Adolescents with Obesity—A Systematic Review

**DOI:** 10.3390/medicina62101851

**Published:** 2026-09-24

**Authors:** Adrianna Zahorowska, Adriana Szczęch, Natalia Lerch, Edyta Śliwak, Yana Osnytska, Aleksandra Marciniak, Andrzej Starczewski, Agnieszka Brodowska, Mariola Krzyścin

**Affiliations:** Department of Gynecology, Endocrinology and Gynecological Oncology, Pomeranian Medical University in Szczecin, 71-252 Szczecin, Poland; a.szczech95@gmail.com (A.S.);

**Keywords:** visceral adipose tissue, children, adolescents, obesity, cardiometabolic risk, body mass index, systematic review

## Abstract

*Background and Objective:* Body mass index (BMI) is widely used to classify obesity in children and adolescents but does not characterize fat distribution. Visceral adipose tissue (VAT) may provide information on cardiometabolic risk beyond BMI. This systematic review aimed to synthesize evidence on the associations between VAT and cardiometabolic risk in children and adolescents with obesity and to evaluate whether VAT provides additional information beyond BMI. *Methods:* PubMed and Scopus were searched for English-language studies published between 1 January 2016 and 28 February 2026. Eligible studies included participants aged 6–19 years, assessed VAT using dual-energy X-ray absorptiometry (DXA), magnetic resonance imaging (MRI), computed tomography (CT), or ultrasonography (USG), and reported at least one cardiometabolic outcome. Two reviewers independently screened the records. Methodological quality was assessed using the appropriate Joanna Briggs Institute Critical Appraisal Tools or the Newcastle–Ottawa Scale. Because of methodological and clinical heterogeneity, the findings were synthesized narratively. The review was reported in accordance with the Preferred Reporting Items for Systematic Reviews and Meta-Analyses (PRISMA) 2020 statement. *Results:* Twenty studies were included, most of which were cross-sectional. Higher VAT was generally associated with insulin resistance, adverse lipid profiles, elevated blood pressure, inflammatory biomarkers, and early vascular dysfunction, although findings were not uniform across all populations and outcomes. In several studies, associations remained significant after adjustment for BMI or other measures of general adiposity, suggesting that VAT may capture cardiometabolic risk not reflected by BMI alone. DXA was the most frequently used assessment method. *Conclusions:* VAT may complement BMI in cardiometabolic risk assessment among children and adolescents with obesity. However, predominantly cross-sectional evidence, methodological heterogeneity, and the absence of standardized pediatric thresholds preclude firm conclusions about its incremental predictive value or routine clinical use. Prospective studies directly comparing VAT and BMI are required.

## 1. Introduction

Pediatric obesity is a major global public health challenge because of its increasing prevalence and substantial health and economic burden [1,2,3]. Obesity during childhood and adolescence frequently persists into adulthood and is associated with an increased risk of type 2 diabetes, cardiovascular disease (CVD), and other non-communicable diseases later in life [4,5]. It is also associated with adverse psychosocial consequences, including depression, low self-esteem, impaired emotional well-being, and reduced quality of life [6].

In pediatric populations, age- and sex-specific body mass index (BMI) is widely used to classify weight status. Obesity is generally defined using age- and sex-specific thresholds derived from standardized growth references, although the applied cutoffs may differ between reference systems [4]. Children and adolescents with obesity are at increased risk of insulin resistance, dyslipidemia, hypertension, metabolic syndrome, and early vascular dysfunction, all of which may contribute to an increased lifetime risk of CVD [7]. Because obesity is a well-established risk factor for cardiometabolic abnormalities and future CVD, children and adolescents with obesity represent a particularly relevant population for early risk assessment and preventive interventions. Although clinically overt cardiovascular disease usually develops in adulthood, subclinical metabolic and vascular alterations associated with atherosclerosis may already be detectable during childhood, particularly among individuals exposed to multiple cardiometabolic risk factors [8].

Despite its widespread clinical use, BMI has important limitations as a measure of adiposity. It does not distinguish fat mass from lean mass and provides no information about regional fat distribution. Consequently, children and adolescents with similar BMI values may differ substantially in body composition and cardiometabolic risk. This heterogeneity is particularly relevant among individuals with obesity, as the metabolic consequences of excess body weight may depend not only on the total amount of adipose tissue but also on where it is deposited [9,10].

Visceral adipose tissue (VAT), defined as adipose tissue located within the abdominal cavity around the internal organs, is a metabolically active fat depot. Greater VAT accumulation has been associated with insulin resistance, dyslipidemia, systemic inflammation, endothelial dysfunction, and other metabolic abnormalities implicated in the development of CVD [9,10]. VAT may therefore provide complementary information on cardiometabolic risk beyond BMI and other measures of general adiposity. In children and adolescents with obesity, its assessment may help distinguish individuals with similar BMI values but different cardiometabolic risk profiles [9,10].

Accurate and standardized assessment of VAT is essential for evaluating its relationship with cardiometabolic risk. Computed tomography (CT) and magnetic resonance imaging (MRI) are generally considered reference methods because they allow direct visualization and compartment-specific quantification of abdominal adipose tissue. However, MRI is limited by cost, examination time, and availability, whereas exposure to ionizing radiation restricts the use of CT for routine assessment and research screening in pediatric populations [11,12].

Dual-energy X-ray absorptiometry (DXA) provides a more practical alternative for body composition assessment. It is a rapid and reproducible imaging method involving a low dose of ionizing radiation and can estimate total and regional body composition, including VAT, using dedicated anatomical regions and software algorithms. DXA-derived VAT estimates have been shown to correlate with measurements obtained using CT and MRI; however, absolute agreement may vary according to the scanner, the software algorithm, anatomical region, participant age, and adiposity level [11,12]. Therefore, VAT measurements obtained using different imaging modalities should not be considered directly interchangeable. Ultrasonography (USG) provides an accessible and radiation-free alternative, but its accuracy may be affected by operator dependence and the lack of standardized pediatric measurement protocols. Overall, DXA and USG may offer feasible approaches for VAT assessment in pediatric research, whereas their role in routine clinical risk assessment remains uncertain.

Despite growing interest in VAT as a marker of metabolic health, evidence concerning its role in children and adolescents with obesity remains fragmented and heterogeneous. Studies differ substantially in participant age, sex, pubertal stage, ethnicity, and obesity severity, as well as in the imaging modalities, anatomical regions, and units used to assess VAT. Considerable variation also exists in the cardiometabolic outcomes evaluated and the covariates included in statistical models. Furthermore, most available studies are cross-sectional, and relatively few have directly assessed whether adding VAT to models based on BMI improves the prediction or classification of cardiometabolic risk. Consequently, the incremental clinical value of VAT beyond BMI remains uncertain.

Therefore, this systematic review aimed to synthesize the available evidence on the associations between imaging-derived VAT and cardiometabolic risk markers in children and adolescents with obesity. A further objective was to examine whether these associations persisted after adjustment for BMI or other measures of general adiposity and whether the available evidence supports an incremental role of VAT in cardiometabolic risk stratification beyond BMI.

## 2. Materials and Methods

### 2.1. Study Design

This systematic review was conducted in accordance with the PRISMA (Preferred Reporting Items for Systematic Reviews and Meta-Analyses) 2020 guidelines [13]. Study selection was conducted in two stages. First, titles and abstracts were screened against the inclusion criteria. Second, full-text reports were assessed for eligibility. Two reviewers conducted the selection independently, resolving disagreements through discussion or consultation with a third reviewer. The review protocol was not prospectively registered in the International Prospective Register of Systematic Reviews (PROSPERO) because registration was not included in the initial protocol-development process. By the time registration was considered, data extraction had already been completed; retrospective registration was therefore not pursued.

### 2.2. Literature Search Strategy

A comprehensive literature search was performed in PubMed and Scopus. These databases were selected a priori because they provide broad and complementary coverage of biomedical and multidisciplinary literature relevant to pediatric obesity, body composition, and cardiometabolic risk. The search was conducted on 28 February 2026 and included studies published between 1 January 2016 and 28 February 2026. This time frame was selected to focus the review on approximately ten years of contemporary evidence reflecting current approaches to VAT assessment and cardiometabolic risk evaluation in pediatric populations.

The search strategy combined terms related to visceral adiposity (e.g., “visceral adipose tissue”, “visceral fat”, “abdominal fat”, and “intra-abdominal fat”), adolescents and youth (e.g., “adolescent”, “teen”, and “youth”), and cardiovascular or cardiometabolic risk (e.g., “cardiovascular risk”, “cardiovascular disease”, “cardiometabolic risk”, “hypertension”, “dyslipidemia”, “insulin resistance”, and “metabolic syndrome”). No sex-related search terms or restrictions were applied. Eligibility screening covered the predefined age range of 6–19 years, and the retrieved records included studies involving both children and adolescents. The complete database-specific search strategies, including the exact queries used in PubMed and Scopus and the number of records retrieved from each database, are presented in Supplementary Table S1.

Data were independently extracted by two reviewers using a standardized extraction form. The extracted information included the author, year of publication, country, study design, sample size, participant characteristics, age, comparator, VAT assessment method, cardiometabolic outcomes, conventional anthropometric measures used for comparison or statistical adjustment, and principal findings. Particular attention was given to analyses evaluating whether associations between VAT and cardiometabolic outcomes persisted after adjustment for BMI or other measures of general adiposity, in accordance with the objective of assessing the potential complementary value of VAT beyond conventional anthropometric measures.

The reference lists of eligible studies were also manually screened to identify potentially relevant publications. Manual screening did not identify any additional records meeting the predefined inclusion criteria.

The inclusion criteria were as follows: (1) studies published in English; (2) children and adolescents aged 6–19 years; (3) inclusion of participants with obesity, either as the primary study population or as a clearly identifiable subgroup; (4) studies including participants with normal weight or overweight when these groups served as comparators or when the study allowed differences in VAT and cardiometabolic risk to be assessed across BMI categories; (5) assessment of visceral adipose tissue using CT, MRI, DXA, or USG; and (6) evaluation of at least one cardiometabolic risk marker, including insulin resistance, dyslipidemia, blood pressure, inflammatory markers, or indicators of cardiovascular dysfunction.

The primary focus of the review was the relationship between VAT and cardiometabolic risk in children and adolescents with obesity. Data from participants without obesity were considered when they provided a comparator for evaluating whether VAT-related cardiometabolic differences could be explained by BMI alone or differed across weight categories.

The exclusion criteria included case reports, conference abstracts without full-text availability, letters to the editor, review articles, studies in which data for participants within the predefined age range could not be evaluated separately, and studies involving participants with genetic disorders, endocrine syndromes (e.g., Cushing syndrome), or chronic diseases significantly affecting metabolism (e.g., cancer). Articles without full-text access were also excluded.

No imputation of missing data was performed. Data extraction was based exclusively on information reported in the original publications, and variables that were unavailable or unclear were recorded as not reported.

The flowchart of the study selection process is presented in Figure 1.

#### PRISMA Process

Following full-text assessment, a total of 713 records were identified from database searches (PubMed and Scopus). Before screening, 277 duplicate records and 44 records for other reasons were removed, leaving 392 records for title and abstract screening. Of these, 332 records were excluded as irrelevant or outside the scope of the review. Consequently, 60 full-text articles were assessed for eligibility. In the eligibility phase, 40 full-text reports were excluded for the following reasons: ineligible age range (n = 5), cardiometabolic risk factors not reported (n = 10), VAT not assessed using an eligible imaging modality (n = 18), and presence of exclusionary comorbidities (n = 7). Ultimately, 20 studies were included in the systematic review.

### 2.3. Quality Assessment

The methodological quality of the included studies was assessed independently by two reviewers using the Joanna Briggs Institute (JBI) Critical Appraisal Tools and the Newcastle–Ottawa Scale (NOS), as appropriate for the respective study designs [14,15]. Any discrepancies were resolved through discussion and consensus. No study was excluded solely on the basis of methodological quality; the appraisal findings were used to inform the interpretation of the evidence. Detailed study-level quality assessments are presented in Supplementary Table S2 and S3.

### 2.4. Data Synthesis

Due to the heterogeneity of study designs, populations, VAT assessment methods, and reported outcomes, a narrative synthesis was performed.

The findings were organized into the following thematic domains:

(a) characteristics of the included studies and methods used for VAT assessment;
(b) the association between VAT accumulation and insulin resistance;
(c) the relationship between VAT and lipid abnormalities;
(d) the association between VAT and blood pressure, vascular dysfunction, and other cardiovascular risk markers;
(e) the role of VAT as a cardiometabolic risk marker beyond BMI and other conventional anthropometric measures.

Within each domain, the direction and consistency of the reported associations were considered, with particular attention to analyses adjusted for BMI or other measures of general adiposity.

Because the included studies reported heterogeneous statistical measures (correlation coefficients, regression coefficients, odds ratios, standardized β coefficients, and group comparisons), no common quantitative effect measure was prespecified, and the original effect estimates were retained and interpreted narratively.

Accordingly, no formal statistical weighting based on sample size was applied. Sample size was considered qualitatively alongside study design, methodological quality, precision, and consistency with the overall evidence. Findings from smaller studies were interpreted more cautiously because of their lower precision and greater susceptibility to random variation. However, larger studies were not automatically considered methodologically superior solely because of their sample size.

### 2.5. Reporting Bias

A formal assessment of reporting bias or publication bias was not performed. This decision was made because the review did not include quantitative meta-analysis, and the substantial methodological and clinical heterogeneity of the included studies precluded meaningful statistical assessment of small-study or publication effects.

### 2.6. Certainty of Evidence

The certainty of the body of evidence was not formally evaluated using the Grading of Recommendations Assessment, Development and Evaluation (GRADE) approach or another certainty framework.

Instead, methodological quality was assessed at the individual study level using design-specific JBI Critical Appraisal Tools and the Newcastle–Ottawa Scale, and these assessments informed the interpretation of the findings.

The completed PRISMA 2020 checklist is provided in Supplementary Table S4.

## 3. Results

### 3.1. Characteristics of Included Studies

A total of 20 studies met the eligibility criteria and were included in the final synthesis [16,17,18,19,20,21,22,23,24,25,26,27,28,29,30,31,32,33,34,35]. Of these, 13 were cross-sectional studies, 2 were cohort studies, 1 was a prospective observational study, 1 was a retrospective observational study, 1 was a pilot study, 1 was a controlled trial, and 1 was a non-randomized controlled intervention study. Sample sizes varied substantially, ranging from 20 to more than 10,000 participants. The included populations comprised children and adolescents aged 6–19 years. The studies were conducted across geographically diverse regions, including North America, South America, Europe, and Asia, with the United States, China, Brazil, and Japan being the most frequently represented countries. The principal characteristics of the included studies are summarized in Table 1. The design-specific JBI Critical Appraisal Tools and Newcastle–Ottawa Scale assessments indicated that most studies were of acceptable methodological quality. The most frequently identified concerns included residual confounding, the inability to establish temporality in cross-sectional studies, and limited sample sizes in several studies. Detailed study-level assessments are provided in Supplementary Table S2 and S3.

### 3.2. Methods Used for VAT Assessment

Several imaging modalities were used to quantify visceral adipose tissue. DXA was used in 12 studies, MRI in 9 studies, CT in 3 studies, and USG in 1 study. Several studies used more than one VAT assessment modality; therefore, the modality counts exceed the total number of included studies.

### 3.3. Cardiometabolic Outcomes Evaluated

The included studies assessed a broad range of cardiometabolic outcomes. The most commonly evaluated parameters included insulin resistance markers, such as the homeostatic model assessment of insulin resistance (HOMA-IR) and fasting insulin levels; lipid profile components, including triglycerides, low-density lipoprotein (LDL) cholesterol, high-density lipoprotein (HDL) cholesterol, and total cholesterol; as well as blood pressure measurements. Several studies additionally investigated inflammatory biomarkers, including high-sensitivity C-reactive protein (hsCRP), leptin, adiponectin, and chemokines. Indicators of early cardiovascular dysfunction, including endothelial function and other vascular parameters, were also evaluated.

A summary of the included studies and their main findings is presented in Table 1.

### 3.4. VAT and Insulin Resistance

Several studies assessed insulin resistance-related outcomes, including HOMA-IR, fasting insulin, and other glucose–insulin markers. In most studies, higher VAT was associated with a less favorable insulin-related profile, most commonly reflected by higher HOMA-IR or fasting insulin concentrations. This association was most evident among children and adolescents with overweight or obesity and, in several studies, remained significant after adjustment for BMI, BMI percentile, pubertal stage, or other measures of general adiposity, suggesting that VAT may capture insulin resistance-related risk not fully reflected by conventional anthropometric parameters [18,22,23,24,33,34]. A similar pattern was observed in female-focused cohorts, in which greater visceral adiposity was linked to higher insulin resistance markers even among girls with normal BMI or after statistical adjustment for BMI-related measures [32,33]. Intervention-based evidence further supported this relationship, as greater reductions in VAT were accompanied by improvements in fasting insulin, HOMA-IR, and insulin sensitivity indices [34]. However, the association between VAT and insulin resistance was not consistent across all pediatric populations. Some studies suggested that this relationship may be weaker in younger individuals, modified by pubertal stage, ethnicity, prenatal metabolic exposure, or birth weight, and partly reflected by broader measures of central or trunk fat distribution rather than VAT alone [20,27,28]. Overall, higher VAT was generally associated with a less favorable insulin-related profile, particularly among children and adolescents with overweight or obesity. However, the consistency and independence of this association varied across populations and statistical adjustment models.

### 3.5. VAT and Lipid Abnormalities

Dyslipidemia was one of the most frequently evaluated cardiometabolic outcomes in the studies included in this review. Most studies demonstrated that higher VAT was associated with a less favorable lipid profile, particularly elevated triglyceride concentrations and lower HDL cholesterol levels. Associations with LDL cholesterol and total cholesterol were also reported, although they were less consistent across studies. Several large cross-sectional studies using DXA demonstrated that increased VAT was independently associated with adverse lipid parameters, suggesting that visceral fat distribution may provide additional information beyond overall adiposity. Similar findings were observed in smaller cohorts, where greater visceral adiposity was linked to a more atherogenic lipid profile and unfavorable lipoprotein characteristics. Intervention studies also demonstrated that reductions in VAT following lifestyle intervention were accompanied by improvements in triglyceride and HDL cholesterol levels, supporting an association between changes in visceral adiposity and lipid profile [31,34]. Taken together, the available evidence indicates that visceral adiposity is associated with dyslipidemia in children and adolescents, particularly elevated triglycerides and reduced HDL cholesterol, and may provide information on metabolic risk not fully reflected by BMI or other conventional anthropometric measures [16,17,18,21,22,23,24,29,33,34,35].

### 3.6. VAT and Blood Pressure and Early Cardiovascular Dysfunction

Several studies evaluated the relationship between VAT and blood pressure or early markers of cardiovascular dysfunction. Most studies reported that greater visceral fat accumulation was associated with higher systolic and/or diastolic blood pressure, particularly among children and adolescents with overweight or obesity [16,21,23,24,35]. Additionally, several studies demonstrated associations between increased VAT and adverse vascular parameters, including measures of vascular and endothelial dysfunction [26]. Collectively, these findings suggest that visceral adiposity may contribute to early cardiovascular alterations before the development of clinically overt cardiovascular disease [16,21,23,24,26]. In several studies, these associations remained significant after adjustment for BMI or waist circumference, indicating that VAT may provide additional information beyond conventional anthropometric measures [16,17,23,24]. However, a small number of studies reported weaker or nonsignificant associations, which may reflect differences in participant characteristics, study design, imaging techniques, or the vascular outcomes evaluated [25]. Taken together, the available evidence suggests that increased VAT is associated with elevated blood pressure and early vascular dysfunction in children and adolescents and may provide complementary information for cardiovascular risk assessment beyond BMI alone [16,17,21,23,24,25,26].

### 3.7. VAT and Inflammatory Biomarkers

Several studies evaluated the association between VAT and inflammatory biomarkers in children and adolescents. Greater visceral adiposity was associated with a more pronounced pro-inflammatory profile, characterized by higher concentrations of hsCRP, leptin, and selected chemokines, as well as lower adiponectin levels. These findings support the concept that visceral adiposity contributes to chronic low-grade inflammation, a key mechanism underlying cardiometabolic dysfunction [18,24,27,28,29,32,33,34,35]. The association between VAT and inflammatory markers was observed primarily in participants with overweight or obesity and, in several studies, remained significant after adjustment for BMI or other measures of adiposity [18,24,33,34]. In addition, intervention studies demonstrated that reductions in VAT were accompanied by improvements in inflammatory parameters, indicating an association between VAT reduction and changes in inflammatory status [34]. Overall, the available evidence generally suggests that increased VAT is associated with an unfavorable inflammatory profile, further supporting its role as an important indicator of cardiometabolic risk beyond conventional measures of obesity.

### 3.8. VAT Beyond BMI and Other Anthropometric Measures

Several studies investigated whether VAT was associated with cardiometabolic risk after adjustment for conventional anthropometric measures, including BMI, waist circumference, and fat mass indices.

In several studies, the associations between VAT and insulin resistance, dyslipidemia, inflammatory biomarkers, blood pressure, and vascular dysfunction remained significant after adjustment for BMI or other anthropometric parameters [16,17,18,21,24,32,33,34]. These findings suggest that visceral fat distribution may capture aspects of metabolically adverse adiposity that are not fully reflected by overall body mass. Furthermore, studies comparing VAT with conventional measures suggested that BMI alone may not fully identify children and adolescents at increased cardiometabolic risk, particularly those with excess visceral fat despite similar BMI values [17,21,32,33]. Taken together, these findings suggest that VAT may provide complementary information for cardiometabolic risk assessment beyond conventional anthropometric measures, particularly in children and adolescents with overweight or obesity.

For each included study, all reported results corresponding to the predefined cardiometabolic outcome domains were considered. When multiple analyses were available, priority was given to multivariable models adjusted for BMI or other measures of general adiposity, as these were most consistent with the objective of examining whether VAT-related associations persisted beyond conventional measures of general adiposity.

The principal findings of the included studies are summarized in Table 2.

## 4. Discussion

### 4.1. Principal Findings

This systematic review synthesized the available evidence on the relationship between VAT and cardiometabolic risk in children and adolescents, particularly those with overweight or obesity. Overall, greater VAT was generally associated with an adverse cardiometabolic profile, including insulin resistance, dyslipidemia, elevated blood pressure, systemic inflammation, and early vascular dysfunction [16,17,18,21,22,23,24,26,29,32,33,34,35]. Importantly, several studies reported that these associations persisted after adjustment for BMI or other measures of general adiposity, suggesting that VAT may capture aspects of cardiometabolic risk not fully reflected by body size alone [16,17,18,21,24,32,33,34]. The evidence was not uniformly consistent. The magnitude and statistical significance of VAT-related associations varied across studies, and some findings suggested that subcutaneous adipose tissue or broader measures of regional fat distribution may be similarly informative for selected outcomes [19,20,25,27,30]. Such heterogeneity may partly reflect differences in age, pubertal stage, sex, ethnicity, VAT assessment methods, and the cardiometabolic outcomes evaluated. This is particularly relevant in pediatric populations, in which body composition, fat distribution, and insulin sensitivity change substantially during growth and puberty [19,27,36]. Taken together, these findings support the concept that VAT may provide complementary information beyond BMI alone in the assessment of cardiometabolic risk in children and adolescents with obesity. However, associations that remain significant after adjustment for BMI do not necessarily demonstrate incremental predictive value. The current evidence therefore does not establish the superiority of VAT over BMI or support its routine use in clinical risk stratification. The clinical relevance of VAT beyond BMI, factors modifying VAT-associated risk, and implications for pediatric risk assessment are discussed below.

### 4.2. VAT as a Marker of Cardiometabolic Risk Beyond BMI

BMI does not account for regional fat distribution, and individuals with similar BMI values may differ substantially in cardiometabolic risk. Several studies included in this review showed that associations between VAT and cardiometabolic abnormalities persisted after adjustment for BMI or other measures of general adiposity. Hatch-Stein et al. reported stronger associations between VAT area and most lipoprotein subclasses than those observed for BMI or HOMA-IR in adolescents with obesity, while van der Heijden et al. demonstrated an adverse metabolic phenotype in girls with normal BMI but increased abdominal adiposity, illustrating that BMI alone may not fully identify metabolically unfavorable fat distribution [17,32]. Further evidence illustrates the potential clinical relevance of discordance between BMI and visceral adiposity. Wang et al. established age- and sex-specific visceral fat area (VFA) cutoffs associated with cardiometabolic risk and showed that even normal-weight boys with VFA above the cutoff had significantly increased cardiometabolic risk [21].

To provide additional context, selected pediatric studies published after the predefined search period or excluded from the primary synthesis are discussed separately below. These studies were not included in the systematic synthesis or methodological quality assessment, and their findings are presented solely as contextual evidence. Cadenas-Sanchez et al., whose study was published after the predefined search date, reported that VAT was associated with clustered cardiometabolic risk in children with overweight or obesity after accounting for other specific fat depots, although the strength of the associations varied across individual outcomes [37]. The study by Higgins et al. was excluded from the primary synthesis because data for participants within the predefined age range could not be evaluated separately; nevertheless, the authors reported an association between VAT and increased pulse wave velocity independent of BMI and waist circumference [38]. These contextual findings are broadly consistent with the results of the primary synthesis but were not used to determine the overall direction or strength of the evidence.

Overall, the findings of the primary synthesis support VAT as a complementary marker rather than a replacement for BMI. Importantly, however, an association that persists after statistical adjustment for BMI does not by itself establish that VAT improves risk prediction when added to BMI-based assessment. Direct prospective comparisons evaluating whether VAT provides incremental discrimination, calibration, or risk reclassification beyond conventional anthropometric measures remain limited. Thus, current evidence supports the biological and potential clinical relevance of VAT beyond BMI, while its incremental value for routine pediatric risk stratification remains to be established.

### 4.3. Factors Modifying VAT-Associated Risk

The relationship between VAT and cardiometabolic risk appears to be influenced by individual characteristics, particularly sex, pubertal stage, and ethnicity. Sex-specific patterns varied across the included studies. Wang et al. reported generally stronger discrimination of VFA for several cardiometabolic outcomes in boys, whereas Hatch-Stein et al. identified several VAT–lipoprotein associations specifically in girls. In contrast, Yan et al. found no significant sex differences in VAT-associated cardiometabolic risk, although sex differences were observed for subcutaneous adipose tissue (SAT) [16,17,21]. Together, these findings suggest that the influence of sex on VAT-associated cardiometabolic risk is not uniform and may depend on the specific outcome assessed. Pubertal stage may represent an additional source of variability, given the substantial changes in body composition and fat distribution occurring during maturation. To contextualize these findings, this and the following sections also refer to additional background evidence that was not included in the primary systematic synthesis or methodological quality assessment. This interpretation is consistent with broader pediatric evidence demonstrating sex- and puberty-related differences in regional fat distribution, with girls generally accumulating more SAT and pubertal and postpubertal boys showing a greater tendency toward abdominal and visceral fat deposition [16,17,36].

Ethnicity may also influence both VAT accumulation and its cardiometabolic significance. Several included studies suggested ethnic differences in regional fat distribution and associated metabolic profiles. Hispanic/Latino youth in some cohorts exhibited greater central adiposity and less favorable metabolic profiles, whereas predominantly African American populations tended to have lower VAT despite substantial overall adiposity [17,27,30,32]. These observations are consistent with previous pediatric evidence showing differences in VAT accumulation among ethnic groups, as well as intervention data demonstrating different amounts of VAT at comparable anthropometric values between Black and White adolescents with obesity [36,39]. Thus, both the distribution and cardiometabolic significance of VAT may vary across ethnic groups, which should be considered when developing and validating pediatric VAT reference values and risk thresholds.

Early-life factors may represent an additional source of heterogeneity. Evidence from studies examining maternal adiposity and other prenatal metabolic exposures suggests that these factors may influence offspring fat distribution and subsequent metabolic risk. Offspring of mothers with overweight or obesity before pregnancy have been shown to exhibit greater visceral fat, lower insulin sensitivity, and a less favorable cardiometabolic profile during childhood. Moreover, maternal prepregnancy BMI contributed independently to offspring insulin sensitivity even after accounting for offspring body fat [40]. However, evidence regarding early-life determinants was limited, and these exposures were not a primary focus of the present review. Collectively, these findings indicate that VAT-associated cardiometabolic risk is heterogeneous and support further development and validation of age-, pubertal stage-, sex-, and ethnicity-specific reference values in pediatric populations.

### 4.4. Clinical Implications and Methods of VAT Assessment

The potential clinical value of VAT assessment depends not only on its association with cardiometabolic risk but also on the feasibility and reliability of the methods used for its measurement. BMI remains the most practical tool for routine assessment of weight status in children and adolescents because of its simplicity, accessibility, and established age- and sex-specific reference standards [4,7]. Therefore, based on the findings of this review, VAT assessment should currently be regarded as complementary to, rather than a replacement for, BMI. Its potential value may lie in providing additional information on fat distribution in individuals whose cardiometabolic risk is not adequately reflected by BMI alone. However, the lack of standardized pediatric VAT thresholds and limited evidence demonstrating incremental predictive value currently preclude its routine use for cardiometabolic risk stratification.

Among the methods represented in the included studies, DXA was the most frequently used technique for VAT assessment. DXA may offer a practical compromise between quantitative assessment and feasibility in pediatric populations. Pediatric validation studies have demonstrated significant correlations between DXA-derived VAT and VAT quantified using CT or MRI [11,12]. Bosch et al. reported that DXA- and CT-derived VAT were significantly correlated and showed broadly similar associations with cardiometabolic risk factors in children [11]. Nevertheless, agreement between techniques is not sufficient to consider them interchangeable. In a longitudinal study of children with normal weight and obesity, DXA-derived VAT was strongly associated with MRI-derived VAT at baseline but showed proportional bias and did not reliably reflect longitudinal changes in MRI-derived VAT [41]. Thus, DXA appears useful for estimating VAT in pediatric research, whereas its role in monitoring changes over time and in individual clinical decision-making requires further validation.

MRI enables detailed quantification of visceral and subcutaneous adipose tissue without exposure to ionizing radiation and is therefore particularly valuable when precise compartment-specific assessment is required. However, its cost, availability, acquisition time, and need for specialized image analysis limit its applicability as a routine screening tool. CT also provides direct assessment of abdominal fat compartments, but exposure to ionizing radiation represents an important limitation in children and makes its use solely for adiposity assessment difficult to justify [11,12,41]. USG may represent a more accessible and radiation-free alternative, although operator dependence, differences in measurement protocols, and the absence of universally accepted pediatric reference values currently limit standardization across studies and clinical settings.

Simpler, low-cost surrogate measures of central adiposity, including waist circumference, waist-to-height ratio (WHtR), waist-to-hip ratio (WHR), and composite indices such as the visceral adiposity index (VAI), may be considered when direct VAT assessment is unavailable. These measures are readily obtainable in clinical practice but do not directly quantify visceral adipose tissue and should not be considered interchangeable with imaging-derived VAT. VAI incorporates BMI, waist circumference, triglycerides, and HDL cholesterol and has been investigated as a marker of metabolic risk. However, pediatric findings regarding its performance have been inconsistent. In one pediatric cohort, VAI was inferior to BMI and waist circumference in its associations with HOMA-IR, inflammatory markers, adipokines, and systolic blood pressure, whereas other studies reported associations between VAI and metabolic syndrome [42,43]. Because these surrogate indices were not systematically evaluated in the present review, their performance cannot be directly compared with imaging-derived VAT on the basis of our findings.

Overall, no single method can currently be recommended for routine VAT-based cardiometabolic risk stratification in children and adolescents. DXA appears to offer a potentially useful balance between feasibility and quantitative assessment, whereas MRI remains valuable when precise characterization of abdominal fat compartments is required. Before VAT assessment can be incorporated into routine pediatric risk stratification, standardized measurement protocols and age-, sex-, and potentially pubertal-specific reference values are needed, together with prospective evidence demonstrating that VAT provides clinically meaningful improvement in risk prediction beyond BMI and other readily available anthropometric measures.

### 4.5. Limitations

Several limitations should be acknowledged. Most included studies had a cross-sectional design, limiting causal inference, and substantial heterogeneity was present in participant characteristics, VAT assessment methods, and cardiometabolic outcomes. Differences in measurement protocols and statistical adjustment, particularly for BMI and other measures of adiposity, further limited direct comparisons across studies and the assessment of the incremental value of VAT beyond BMI. The substantial variation in sample size across the included studies should also be considered when interpreting the findings. Estimates from smaller studies may be less precise and more susceptible to random variation, whereas sample size alone does not determine overall methodological quality. The restriction of the literature search to PubMed and Scopus represents a major limitation of this review. Although these databases provide broad coverage of biomedical and multidisciplinary literature, limiting the search to two databases may have reduced the comprehensiveness of the evidence retrieved and may have resulted in relevant studies being missed. The search was additionally restricted to English-language publications and studies published between 1 January 2016 and 28 February 2026, which may have further limited the retrieval of relevant evidence. In addition, the population component of the search relied primarily on adolescent- and youth-related terminology, which may have reduced search sensitivity for studies focusing exclusively on younger children. The lack of prospective PROSPERO registration represents an additional methodological limitation because it limits transparency regarding the prespecified review methods and any deviations from the original protocol. Studies published after the predefined search period or otherwise excluded from the primary synthesis were cited solely for contextual purposes and were not included in the systematic synthesis or methodological quality assessment. Substantial methodological and clinical heterogeneity across studies precluded quantitative meta-analysis and necessitated a narrative synthesis. Accordingly, although the available evidence supports an association between VAT and cardiometabolic risk beyond general adiposity, it does not establish incremental predictive value or clinically applicable pediatric VAT thresholds. In addition, publication and reporting bias were not formally assessed. Consequently, the possibility that studies with null or unfavorable findings remain underrepresented cannot be excluded. Similarly, the certainty of the overall body of evidence was not formally graded. Therefore, the conclusions should be interpreted in light of the predominance of observational, largely cross-sectional evidence.

## 5. Conclusions

The available evidence suggests that visceral adipose tissue is associated with insulin resistance, dyslipidemia, inflammation, elevated blood pressure, and early cardiovascular dysfunction in children and adolescents, particularly those with obesity. Compared with BMI alone, VAT may provide complementary information regarding cardiometabolic risk; however, its incremental predictive value and clinical utility for pediatric risk stratification remain to be established. DXA appears to be a practical method for VAT assessment, although further prospective studies are needed to establish standardized pediatric VAT thresholds and to determine whether VAT improves prediction of long-term cardiometabolic outcomes beyond conventional anthropometric measures.

## Figures and Tables

**Figure 1:**
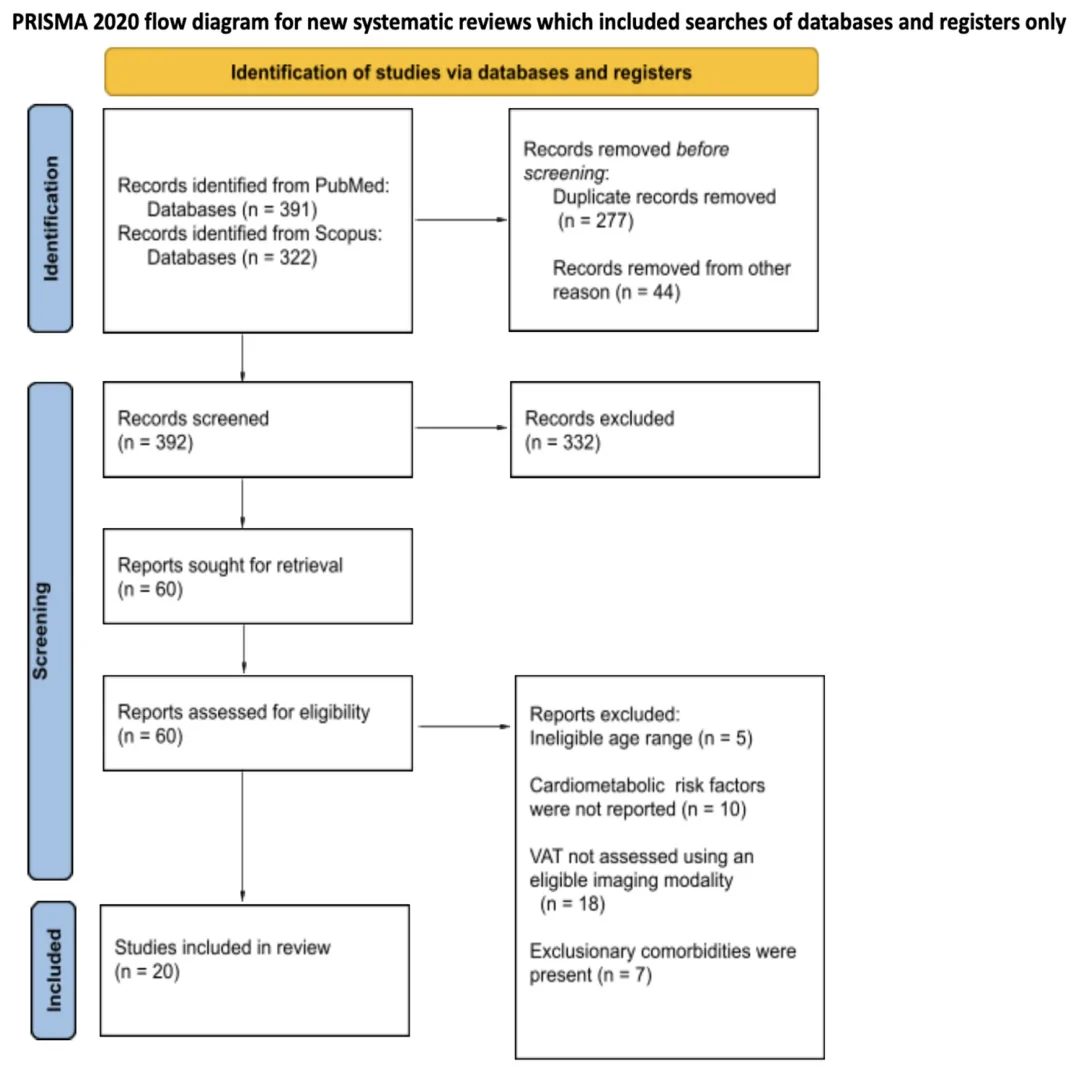
PRISMA flow diagram illustrating the study selection process for the systematic review: Beyond body mass index: the role of visceral adiposity in cardiometabolic risk stratification among children and adolescents with obesity.

**Table 1:** Characteristics and main findings of studies included in the systematic review.

Study (Country)	Design and Participants	VAT Assessment	Groups/Intervention	Outcomes and Other Measures	Main Finding
Yan et al. (2019) [16] (China)	Cross-sectional; F: n = 4193; M: n = 4267; age 6–18 years	DXA	Normal weight, overweight, obesity	BP, LDL, HDL, TC, TG; SAT, BMI, FMI	VAT and SAT may identify young people with overweight or obesity at increased cardiovascular risk.
Hatch-Stein et al. (2016) [17] (USA)	Cross-sectional; F: n = 68; M: n = 59; age 12–18 years	DXA	—	HDL, LDL, VLDL-P, TG, HOMA-IR; BMI, WC	VAT area was associated with atherogenic lipoproteins and provided information beyond BMI and HOMA-IR; the LDL association differed by sex.
Wu et al. (2021) [18] (USA)	Cross-sectional; F: n = 374; M: n = 366; mean age 13 years	DXA	—	SBP, HDL, TG, HOMA-IR, ALT, hsCRP, leptin, adiponectin; SAAT, TAAT, BMI	Abdominal adiposity was associated with adverse cardiometabolic markers, partly independently of BMI.
Hübers et al. (2017) [19] (Germany)	Cross-sectional; F: n = 91; M: n = 64; age 6–18 years	MRI	Derivation and cross-validation groups	BP, TG, HDL, HOMA-IR; FM, FFM, BMI, WC	Associations were weak before and during puberty but stronger after puberty.
Grunnet et al. (2017) [20] (Denmark)	Cohort; n = 546–561; F: 47.4%; age 9–16 years	DXA	Control: n = 597; F: 49.6%	TC, LDL, HDL, TG, C-peptide, HOMA-IR; BMI, WC, maternal prepregnancy BMI	After adjustment for offspring BMI, DXA body-composition measures did not differ significantly between groups.
Wang et al. (2025) [21] (China)	Cross-sectional with longitudinal validation; cohort 1, F: n = 4030; cohort 2, F: n = 5453; age 6–18 years	DXA	Validation cohort: n = 10,805	BP, TC, TG, LDL, HDL; BMI, BIA, VFA	Higher VFA was associated with cardiometabolic risk; age- and sex-specific cutoffs also identified risk in normal-weight boys.
Eloi et al. (2017) [22] (Brazil)	Prospective observational; group A, F: n = 16; group B, F: n = 13; age 16–18 years	MRI	Healthy vs. overweight/obesity	BP, TC, LDL, HDL, TG, HOMA-IR; body surface area, WC, BMI	Visceral fat percentage was associated with TG in both groups, significantly only in the overweight/obesity group.
Syme et al. (2016) [23] (Canada)	Cohort; F: n = 511; M: n = 479; age 12–18 years	MRI	—	BP, insulin, TG, HDL; BMI, WC	Visceral fat was positively associated with BP, insulin, and TG.
Kelly et al. (2020) [24] (USA)	Cross-sectional; F: n = 297; M: n = 260; age 8–18 years	DXA	—	BP, TG, LDL, HDL, insulin, hsCRP, HMW adiponectin; %BF, BMI	Above the identified threshold, VAT was associated with most cardiometabolic risk factors.
Fearnbach et al. (2020) [25] (USA)	Pilot; F: n = 11; M: n = 9; age 11–15 years	DXA, MRI	—	CRF, BP, HOMA-IR, LDL, TG, hsCRP, risk score; BMI	VAT was not associated with cardiometabolic markers; VO_2_peak was inversely associated with SAT but not VAT.
Tomsa et al. (2016) [26] (USA)	Cross-sectional; F: n = 46; M: n = 35; age 12–19 years	DXA, MRI	Normal-weight controls: M, n = 12; F, n = 9	Augmentation index, TC, LDL, HDL, TG, BP, leptin; BMI	RHI was inversely associated with fat mass, body-fat percentage, abdominal fat, and BMI indices.
Martos-Moreno et al. (2020) [27] (Spain)	Cross-sectional; F: n = 75; M: n = 75; pubertal: n = 100; prepubertal: n = 50; age 11–16 years	DXA, MRI	Caucasian vs. Latino	Leptin, adiponectin, HOMA-IR, LDL, HDL, TG; BMI, liver USG, T	Latino children with obesity had greater trunk-fat accumulation linked to metabolic disturbances and liver steatosis.
Stansfield et al. (2016) [28] (USA)	Cross-sectional; F: n = 299; M: n = 276; age 14–18 years	MRI	Birth-weight tertiles	Leptin, adiponectin, HOMA-IR, LDL, HDL, TG, BP, hsCRP; BMI	Low and high birth weight were associated with greater VAT, which may indicate CVD risk earlier than conventional lipids.
Ishii et al. (2016) [29] (Japan)	Cross-sectional; F: n = 18; M: n = 26; age 6.8–16.5 years	CT	Chemokine levels	FPG, insulin, ALT, adiponectin, HOMA-IR, LDL, HDL, TG; BMI, WC	CXCL10 correlated positively with VFA, ALT, UA, and LDL-C and negatively with FPG; CCL2 was elevated in metabolic syndrome.
Duran et al. (2020) [30] (USA)	Retrospective observational; F: n = 2629; M: n = 3909; age 8–19 years	DXA	Three ethnicity groups	TG, LDL; T2L, FMI, BMI	In females, LDL-C and TG correlated positively with FMI and T2L z-scores.
de Souza Marques et al. (2023) [31] (Brazil)	Controlled trial; F: n = 23; M: n = 20; age 11–16 years	MRI, CT	Family vs. individual 12-week lifestyle intervention	BP, HDL, TG, TC, HOMA-IR, LDL; BMI, WC	The intervention improved adiposity and cardiometabolic risk in adolescents with overweight or obesity.
van der Heijden et al. (2018) [32] (Spain)	Cross-sectional; F: n = 46; age 13–17 years	DXA, MRI	Normal BMI with high vs. normal fat content	HOMA-IR, leptin, hsCRP, WBISI, adiponectin, fasting insulin; BMI	In sedentary Hispanic girls with normal BMI, higher VAT indicated metabolic risk not captured by BMI.
Bland et al. (2022) [33] (USA)	Cross-sectional; F: n = 337; age 9–12 years	DXA	Non-Hispanic: n = 92; Hispanic: n = 245; BMI groups	LDL, HDL, HOMA-IR, TG, CRP, BP; BMI	Adjusted VAT was associated with higher TG in girls with overweight/obesity and higher HOMA-IR in both BMI groups.
Campos et al. (2019) [34] (Brazil)	Non-randomized trial; F: n = 102; M: n = 70; age 15–19 years	USG	Exercise, nutrition counseling, physiotherapy	LDL, HDL, HOMA-IR, TG, hsCRP, BP, leptin; BMI	Reduced VAT was associated with improved metabolic markers and often outperformed BMI as a marker of improvement.
Yasuda et al. (2021) [35] (Japan)	Cross-sectional; T2D: n = 19 (M: 53%); MS: n = 19 (M: 78.9%); healthy: n = 69 (M: 43%); age 7–17 years	CT	T2D, MS, neither	LDL, HDL, TG, BP, leptin; BMI, WC	VAT accumulation was linked to insulin resistance and complications including MS, NAFLD, and T2D.

Abbreviations: %BF, body fat percentage; ALT, alanine aminotransferase; BIA, bioelectrical impedance analysis; BMI, body mass index; BP, blood pressure; CCL2, C-C motif chemokine ligand 2; CRF, cardiorespiratory fitness; CRP, C-reactive protein; CT, computed tomography; CVD, cardiovascular disease; CXCL10, C-X-C motif chemokine ligand 10; DXA, dual-energy X-ray absorptiometry; F, female; FFM, fat-free mass; FM, fat mass; FMI, fat mass index; FPG, fasting plasma glucose; HDL, high-density lipoprotein; HMW, high-molecular-weight adiponectin; HOMA-IR, homeostatic model assessment of insulin resistance; hsCRP, high-sensitivity C-reactive protein; LDL, low-density lipoprotein; LDL-C, low-density lipoprotein cholesterol; M, male; MRI, magnetic resonance imaging; MS, metabolic syndrome; NAFLD, non-alcoholic fatty liver disease; RHI, reactive hyperemia index; SAAT, subcutaneous abdominal adipose tissue; SAT, subcutaneous adipose tissue; SBP, systolic blood pressure; T2D, type 2 diabetes; T2L, trunk-to-leg fat ratio; TAAT, total abdominal adipose tissue; TC, total cholesterol; TG, triglycerides; UA, uric acid; USG, ultrasonography; VAT, visceral adipose tissue; VFA, visceral fat area; VLDL-P, very-low-density lipoprotein particles; VO_2_peak, peak oxygen uptake; WBISI, whole-body insulin sensitivity index; WC, waist circumference.

**Table 2:** Summary of the Main Findings Regarding the Association Between Visceral Adipose Tissue and Cardiometabolic Risk in Children and Adolescents With Obesity.

Domain	Main Findings	Potential Clinical Implication
Insulin resistance	Higher VAT was generally associated with increased insulin resistance, reflected by elevated HOMA-IR and fasting insulin concentrations. Several studies reported that these associations remained significant after adjustment for BMI.	VAT assessment may improve the identification of adolescents at increased risk of insulin resistance beyond conventional anthropometric measures.
Lipid abnormalities	Greater VAT was primarily associated with elevated triglyceride concentrations and lower HDL cholesterol levels, whereas associations with LDL cholesterol and total cholesterol were less consistent.	VAT may provide additional information regarding obesity-related dyslipidemia beyond BMI alone.
Blood pressure and early cardiovascular dysfunction	Increased VAT was associated with elevated blood pressure and adverse vascular changes, including impaired endothelial and vascular function.	Assessment of VAT may facilitate the early identification of adolescents at increased cardiovascular risk.
Inflammatory biomarkers	Higher VAT was associated with a more adverse inflammatory profile, characterized by increased hsCRP and leptin concentrations together with lower adiponectin levels.	VAT may reflect a metabolically active adipose tissue contributing to chronic low-grade inflammation and cardiometabolic dysfunction.
Risk stratification beyond BMI	Most included studies demonstrated that VAT remained associated with cardiometabolic abnormalities after adjustment for BMI or other conventional anthropometric measures.	Incorporating VAT assessment into the clinical evaluation of adolescents with obesity may improve cardiometabolic risk stratification beyond BMI alone.

## Data Availability

No new data were created or analyzed in this study. Data sharing is not applicable to this article.

## Supplementary Materials

The following supporting information can be downloaded at: https://www.mdpi.com/article/10.3390/medicina62101851/s1, Table S1: Complete database-specific search strategies for PubMed and Scopus; Table S2: Methodological quality assessment using the Joanna Briggs Institute (JBI) Critical Appraisal Tools; Table S3: Methodological quality assessment using the Newcastle–Ottawa Scale (NOS). Table S4: PRISMA 2020 Checklist.

## Abbreviations

The following abbreviations are used in this manuscript:

BIA	Bioelectrical impedance analysis
BMI	Body mass index
CMR	Cardiometabolic risk
CT	Computed tomography
CVD	Cardiovascular disease
DXA	Dual-energy X-ray absorptiometry
HDL	High-density lipoprotein
HOMA-IR	Homeostatic Model Assessment of Insulin Resistance
JBI	Joanna Briggs Institute
LDL	Low-density lipoprotein
MRI	Magnetic resonance imaging
NOS	Newcastle–Ottawa Scale
PRISMA	Preferred Reporting Items for Systematic Reviews and Meta-Analyses
SAT	Subcutaneous adipose tissue
USG	Ultrasonography
VAI	Visceral Adiposity Index
VAT	Visceral adipose tissue
VFA	Visceral fat area
