# Supplementary material for: Beyond BMI: The Role of Visceral Adipose Tissue in Cardiometabolic Risk Stratification Among Children and Adolescents with Obesity—A Systematic Review
Source: Medicina (Kaunas). 2026 Sep 24;62(10):1851. doi: 10.3390/medicina62101851 (PMC13707073; doi:10.3390/medicina62101851)
Supplement: Supplementary file 1 [file medicina-62-01851-s001.zip › Table S1 NOS_Risk_of_Bias_Beyond_BMI_VAT_Systematic_Review.pdf]

Beyond BMI: The Role of Visceral Adipose Tissue in Cardiometabolic Risk Stratification Among Children and Adolescents with Obesity — A Systematic Review

Methodological approach

The original Newcastle-Ottawa Scale (NOS) is intended for non-randomized cohort and case-control studies. Accordingly, the standard 9-star cohort NOS was applied to longitudinal cohort analyses. Because most included observational publications used cross-sectional analytical designs, these studies were evaluated using an explicitly adapted Newcastle-Ottawa Scale for cross-sectional studies (maximum 10 stars), preserving the NOS domains of Selection, Comparability, and Outcome. The adapted version assessed sample representativeness, sample-size justification, non-response, exposure ascertainment, control of confounding, outcome assessment, and statistical analysis. Randomized and quasi-experimental intervention studies were not assigned NOS star scores because doing so would misapply the instrument.

Interpretation of star totals: The Newcastle-Ottawa Scale does not provide universally accepted official cut-offs for “low”, “moderate”, or “high” risk of bias. Therefore, the overall methodological appraisal below is domain-based. Descriptive labels are used to summarize the pattern of concerns and should not be interpreted as validated NOS thresholds.

Summary of risk-of-bias assessment

Study	Study design	NOS approach	Total score	Overall appraisal
Kelly et al.	Analytical cross-sectional	Adapted NOS (cross-sectional)	8/10	Lower methodological concerns
Wu et al.	Analytical cross-sectional	Adapted NOS (cross-sectional)	8/10	Lower methodological concerns
Tomsa et al.	Analytical cross-sectional	Adapted NOS (cross-sectional)	7/10	Moderate methodological concerns
Stansfield et al.	Analytical cross-sectional	Adapted NOS (cross-sectional)	7/10	Moderate methodological concerns
Syme et al.	Analytical cross-sectional	Adapted NOS (cross-sectional)	8/10	Lower methodological concerns
Martos-Moreno et al.	Analytical cross-sectional	Adapted NOS (cross-sectional)	8/10	Lower methodological concerns
van der Heijden et al.	Analytical cross-sectional	Adapted NOS (cross-sectional)	6/10	Moderate methodological concerns
Duran et al.	Analytical cross-sectional	Adapted NOS (cross-sectional)	8/10	Lower methodological concerns
Hatch-Stein et al.	Analytical cross-sectional	Adapted NOS (cross-sectional)	8/10	Lower methodological concerns

Study	Study design	NOS approach	Total score	Overall appraisal
Eloi et al.	Analytical cross-sectional	Adapted NOS (cross-sectional)	6/10	Moderate methodological concerns
Hübers et al.	Analytical cross-sectional	Adapted NOS (cross-sectional)	8/10	Lower methodological concerns
Ishii et al.	Analytical cross-sectional	Adapted NOS (cross-sectional)	6/10	Moderate methodological concerns
Fearnbach et al.	Analytical cross-sectional	Adapted NOS (cross-sectional)	7/10	Moderate methodological concerns
Bland et al.	Analytical cross-sectional	Adapted NOS (cross-sectional)	8/10	Lower methodological concerns
Yan et al.	Analytical cross-sectional	Adapted NOS (cross-sectional)	8/10	Lower methodological concerns
Yasuda et al.	Analytical cross-sectional	Adapted NOS (cross-sectional)	6/10	Moderate methodological concerns
Grunnet et al.	Longitudinal cohort	Standard NOS (cohort)	8/9	Lower methodological concerns
Wang et al.	Longitudinal cohort	Standard NOS (cohort)	8/9	Lower methodological concerns
de Souza Marques et al.	Randomized controlled trial	NOS not applicable	N/A	JBI tool needed
Campos et al.	Quasi-experimental pre-post study	NOS not applicable	N/A	JBI quasi-experimental tool needed
