# Supplementary material for: Beyond BMI: The Role of Visceral Adipose Tissue in Cardiometabolic Risk Stratification Among Children and Adolescents with Obesity—A Systematic Review
Source: Medicina (Kaunas). 2026 Sep 24;62(10):1851. doi: 10.3390/medicina62101851 (PMC13707073; doi:10.3390/medicina62101851)
Supplement: Supplementary file 1 [file medicina-62-01851-s001.zip › Table S2 JBI_Critical_Appraisal_Beyond_BMI_VAT_Systematic_Review.pdf]

Beyond BMI: The Role of Visceral Adipose Tissue in Cardiometabolic Risk Stratification Among Children and Adolescents with Obesity — A Systematic Review

Methodological approach

Critical appraisal was performed using design-specific Joanna Briggs Institute (JBI) tools. Analytical cross-sectional studies were appraised with the JBI tool for analytical cross-sectional studies; longitudinal cohort analyses with the JBI cohort tool; the randomized intervention study with the JBI randomized controlled trial tool; and the non-randomized pre-post intervention study with the JBI quasi-experimental tool. No case report or case series was identified among the 20 uploaded publications. Judgements were recorded as Yes, No, Unclear, or Not Applicable (N/A).

No arbitrary numerical quality score or percentage threshold was imposed. In keeping with contemporary JBI methodology, the appraisal is domain-based and the overall interpretation is descriptive, with particular attention to selection, measurement, confounding, temporal inference, participant retention, and statistical conclusion validity.

Summary of study designs and overall methodological appraisal

Study	Design	JBI tool	Yes	No	Unclear	N/A	Overall methodological appraisal
Kelly et al. (2020)	Analytical cross-sectional study	Analytical Cross-Sectional Studies	6	0	1	1	Acceptable methodological quality with some concerns related to pooled-source selection and the cross-sectional design.
Wu et al. (2021)	Analytical cross-sectional study	Analytical Cross-Sectional Studies	7	0	0	1	Generally robust for an analytical cross-sectional study; residual confounding and lack of

Study	Design	JBI tool	Yes	No	Unclear	N/A	Overall methodological appraisal
							temporality remain inherent limitations.
Tomsa et al. (2016)	Analytical cross-sectional study	Analytical Cross-Sectional Studies	8	0	0	0	Generally robust analytical cross-sectional evidence, with the principal limitation being inability to establish temporal or causal relationships.
Stansfield et al. (2016)	Analytical cross-sectional study	Analytical Cross-Sectional Studies	6	0	1	1	Acceptable methodological quality with concern regarding retrospective parental recall of birth weight and cross-sectional outcome assessment.
Syme et al. (2016)	Analytical cross-sectional study	Analytical Cross-Sectional Studies	7	0	0	1	Generally robust cross-sectional analysis within a cohort; causal direction cannot be established from the

**Study**	**Design**	**JBI tool**	**Yes**	**No**	**Unclear**	**N/A**	**Overall methodological appraisal**
							contemporaneous measurements.
de Souza Marques et al. (2023)	Randomized controlled trial	Randomized Controlled Trials	7	5	1	0	Methodological concerns are driven primarily by absent allocation concealment, unavoidable lack of participant/provider blinding, and incomplete per-protocol follow-up.
Martos-Moreno et al. (2020)	Analytical cross-sectional study	Analytical Cross-Sectional Studies	8	0	0	0	Generally robust analytical cross-sectional study; residual confounding and cross-sectional temporality remain relevant.
van der Heijden et al. (2018)	Analytical cross-sectional study	Analytical Cross-Sectional Studies	6	2	0	0	Substantial methodological concerns arise from the very small sample and lack of multivariable control for confounding.

Study	Design	JBI tool	Yes	No	Unclear	N/A	Overall methodological appraisal
Duran et al. (2020)	Analytical cross-sectional study	Analytical Cross-Sectional Studies	7	0	0	1	Generally acceptable methodological quality; interpretation is limited by the retrospective cross-sectional design and by trunk/leg ratio being a surrogate rather than direct VAT measurement.
Hatch-Stein et al. (2016)	Analytical cross-sectional study	Analytical Cross-Sectional Studies	8	0	0	0	Generally robust analytical cross-sectional study, although generalizability is limited by the predominantly African-American sample.
Eloi et al. (2017)	Analytical cross-sectional study	Analytical Cross-Sectional Studies	6	1	1	0	Acceptable exploratory study with important concerns regarding small sample size and limited control of confounding.

Study	Design	JBI tool	Yes	No	Unclear	N/A	Overall methodological appraisal
Grunnet et al. (2017)	Cohort study	Cohort Studies	7	0	4	0	Moderate methodological concerns, principally related to exposure ascertainment and potential selection from long-term cohort attrition.
Hübers et al. (2017)	Analytical cross-sectional study	Analytical Cross-Sectional Studies	7	0	1	0	Acceptable methodological quality; pooled cross-sectional data limit causal inference and introduce some uncertainty regarding sample selection.
Ishii et al. (2016)	Analytical cross-sectional study	Analytical Cross-Sectional Studies	6	2	0	0	Substantial concerns relate to small sample size, absence of lean controls, and lack of multivariable confounder adjustment.
Campos et al. (2019)	Quasi-experimental pre-post study	Quasi-Experimental Studies	7	2	0	0	Moderate methodological concerns because the

Study	Design	JBI tool	Yes	No	Unclear	N/A	Overall methodological appraisal
							study lacked a concurrent control group and relied on only one pre- and one post-intervention assessment.
Fearnbach et al. (2020)	Analytical cross-sectional study	Analytical Cross-Sectional Studies	8	0	0	0	Acceptable pilot evidence, but precision and generalizability are limited by the very small analytic sample.
Bland et al. (2022)	Analytical cross-sectional study	Analytical Cross-Sectional Studies	7	0	0	1	Generally robust analytical cross-sectional study, with residual confounding and limited causal inference inherent to the design.
Wang et al. (2025)	Cross-sectional derivation study with longitudinal cohort validation	Cohort Studies (applied to the longitudinal validation component, while considering the cross-sectional derivation phase)	10	0	1	0	Generally strong large-sample evidence for short-term risk stratification, with uncertainty regarding baseline outcome-free

Study	Design	JBI tool	Yes	No	Unclear	N/A	Overall methodological appraisal
							status and limited representativeness from nonrandom sampling.
Yan et al. (2019)	Analytical cross-sectional study	Analytical Cross-Sectional Studies	7	0	0	1	Generally robust analytical cross-sectional evidence, with the principal limitation being inability to determine temporality or causality.
Yasuda et al. (2021)	Analytical cross-sectional study	Analytical Cross-Sectional Studies	6	1	1	0	Acceptable exploratory marker study with important concerns regarding confounding and the cross-sectional comparison of established clinical groups.

Detailed study-level appraisals

1. Kelly et al. (2020)

Reaching the Tipping Point: Identification of Thresholds at which Visceral Adipose Tissue May Steeply Increase in Youth

Study design: Analytical cross-sectional study **Appraisal tool:** JBI Critical Appraisal Tool for Analytical Cross-Sectional Studies

No.	JBI appraisal criterion	Judgement	Scientific justification
1	Were the criteria for inclusion in the sample clearly defined?	Unclear	The analysis combined participants from four parent studies with identical measurement procedures, but a single harmonized set of eligibility and exclusion criteria for the pooled analytical sample was not fully specified.
2	Were objective, standard criteria used for measurement of the condition?	N/A	Participants were recruited across a wide BMI range rather than on the basis of a single disease diagnosis or condition requiring standardized diagnostic criteria; this item was therefore considered not applicable.
3	Was the exposure measured in a valid and reliable way?	Yes	Visceral adipose tissue was estimated by DXA using standardized procedures. Although MRI/CT are reference methods, the DXA approach was described and supported by validation evidence.
4	Were the outcomes measured in a valid and reliable way?	Yes	Cardiometabolic outcomes were obtained using standardized fasting laboratory measurements and standardized blood-pressure procedures.
5	Were confounding factors identified?	Yes	Relevant potential confounders and effect modifiers, including sex, age, race/ethnicity, pubertal

No.	JBI appraisal criterion	Judgement	Scientific justification
			stage, and overall adiposity, were considered.
6	Were strategies to deal with confounding factors stated?	Yes	Segmented and linear regression models incorporated prespecified adjustment, including Tanner stage and demographic stratification.
7	Was appropriate statistical analysis used?	Yes	Segmented regression and robust variance estimation were appropriate for the threshold-based research question.
8	Were the study subjects and the setting described in detail?	Yes	Age range, geographic recruitment area, BMI distribution, measurement procedures, and participant characteristics were reported in sufficient detail.

Overall methodological appraisal: Acceptable methodological quality with some concerns related to pooled-source selection and the cross-sectional design.

2. Wu et al. (2021)

Associations of DXA-measured abdominal adiposity with cardio-metabolic risk and related markers in early adolescence in Project Viva

Study design: Analytical cross-sectional study **Appraisal tool:** JBI Critical Appraisal Tool for Analytical Cross-Sectional Studies

No.	JBI appraisal criterion	Judgement	Scientific justification
1	Were the criteria for inclusion in the sample clearly defined?	Yes	The analytical sample was drawn from Project Viva with clearly described adolescent participants and availability of DXA and cardiometabolic measurements.
2	Were objective, standard criteria used for measurement of the condition?	N/A	The study evaluated a community-based cohort rather than selecting participants according to a specific disease diagnosis; this criterion was not applicable.
3	Was the exposure measured in a valid and reliable way?	Yes	VAT, SAAT, and total abdominal adipose tissue were estimated by DXA using a standardized acquisition and analytic protocol.
4	Were the outcomes measured in a valid and reliable way?	Yes	Cardiometabolic risk components, inflammatory markers, adipokines, blood pressure, and biochemical outcomes were measured using established procedures.
5	Were confounding factors identified?	Yes	The authors identified major confounders including age, sex, race/ethnicity, pubertal status, and BMI z-score.
6	Were strategies to deal with confounding factors stated?	Yes	Multivariable linear regression models explicitly adjusted for the identified confounders and included sex-stratified analyses.
7	Was appropriate statistical analysis used?	Yes	The regression approach and transformations were appropriate for the continuous outcomes and study objectives.

No.	JBI appraisal criterion	Judgement	Scientific justification
8	Were the study subjects and the setting described in detail?	Yes	The source cohort, age, sex distribution, geographic setting, and key participant characteristics were clearly described.

Overall methodological appraisal: Generally robust for an analytical cross-sectional study; residual confounding and lack of temporality remain inherent limitations.

3. Tomsa et al. (2016)

Endothelial Function in Youth: A Biomarker Modulated by Adiposity-Related Insulin Resistance

Study design: Analytical cross-sectional study **Appraisal tool:** JBI Critical Appraisal Tool for Analytical Cross-Sectional Studies

No.	JBI appraisal criterion	Judgement	Scientific justification
1	Were the criteria for inclusion in the sample clearly defined?	Yes	Eligibility and comparison groups were clearly defined across normal-weight, overweight, impaired-glucose-regulation, and type 2 diabetes categories.
2	Were objective, standard criteria used for measurement of the condition?	Yes	Weight and glycemic categories were established using objective anthropometric and metabolic criteria.
3	Was the exposure measured in a valid and reliable way?	Yes	Body composition and abdominal fat partitioning were assessed by DXA and abdominal MRI using standardized methods.
4	Were the outcomes measured in a valid and reliable way?	Yes	Endothelial function, insulin sensitivity, glucose tolerance, lipids, and inflammatory biomarkers were assessed with validated or objective techniques, including peripheral arterial tonometry and the hyperinsulinemic-euglycemic clamp.
5	Were confounding factors identified?	Yes	Age, sex, race/ethnicity, adiposity, insulin sensitivity, and glycemic status were considered as potential explanatory or confounding factors.
6	Were strategies to deal with confounding factors stated?	Yes	Multiple regression models were used to examine independent determinants of vascular outcomes.

No.	JBI appraisal criterion	Judgement	Scientific justification
7	Was appropriate statistical analysis used?	Yes	Correlation, group-comparison, and multivariable regression methods were appropriate to the analytical objectives.
8	Were the study subjects and the setting described in detail?	Yes	The four study groups, clinical setting, sample size, demographics, and measurement procedures were adequately reported.

Overall methodological appraisal: Generally robust analytical cross-sectional evidence, with the principal limitation being inability to establish temporal or causal relationships.

4. Stansfield et al. (2016)

Nonlinear Relationship between Birth Weight and Visceral Fat in Adolescents

Study design: Analytical cross-sectional study **Appraisal tool:** JBI Critical Appraisal Tool for Analytical Cross-Sectional Studies

No.	JBI appraisal criterion	Judgement	Scientific justification
1	Were the criteria for inclusion in the sample clearly defined?	Yes	Age, race, medication, and medical-condition eligibility criteria were explicitly reported.
2	Were objective, standard criteria used for measurement of the condition?	N/A	Participants were not recruited on the basis of a disease diagnosis; standardized diagnostic classification was not central to eligibility.
3	Was the exposure measured in a valid and reliable way?	Unclear	The principal etiologic exposure, birth weight, was obtained by parental recall rather than medical records. Although prior validation studies were cited, recall error remains possible.
4	Were the outcomes measured in a valid and reliable way?	Yes	Adolescent VAT, anthropometry, blood pressure, biochemical markers, and related outcomes were assessed using standardized objective procedures.
5	Were confounding factors identified?	Yes	The study identified important confounding factors including sex, race, socioeconomic status, pubertal stage, physical activity, and adiposity.
6	Were strategies to deal with confounding factors stated?	Yes	Adjusted analyses were undertaken to account for relevant covariates and potential confounders.
7	Was appropriate statistical analysis used?	Yes	The nonlinear/tertile-based analyses and adjusted comparisons were broadly appropriate, although

No.	JBI appraisal criterion	Judgement	Scientific justification
			categorization of birth weight may reduce information.
8	Were the study subjects and the setting described in detail?	Yes	Recruitment from local high schools, the Augusta setting, age/race composition, and measurement period were clearly reported.

Overall methodological appraisal: Acceptable methodological quality with concern regarding retrospective parental recall of birth weight and cross-sectional outcome assessment.

5. Syme et al. (2016)

Glycerophosphocholine Metabolites and Cardiovascular Disease Risk Factors in Adolescents: A Cohort Study

Study design: Analytical cross-sectional study **Appraisal tool:** JBI Critical Appraisal Tool for Analytical Cross-Sectional Studies

No.	JBI appraisal criterion	Judgement	Scientific justification
1	Were the criteria for inclusion in the sample clearly defined?	Yes	The population-based Saguenay Youth Study sample and eligibility framework were described sufficiently for the analytical sample.
2	Were objective, standard criteria used for measurement of the condition?	N/A	The analysis was not based on a disease-defined sample; therefore, this item was not applicable.
3	Was the exposure measured in a valid and reliable way?	Yes	Visceral fat was measured by MRI and circulating glycerophosphocholines by targeted mass spectrometry using standardized laboratory procedures.
4	Were the outcomes measured in a valid and reliable way?	Yes	Blood pressure, insulin resistance, fasting lipids, and other cardiometabolic outcomes were assessed using objective standardized measures.
5	Were confounding factors identified?	Yes	Potential confounding by age, sex, adiposity, and other cardiometabolic factors was considered in the analytical framework.
6	Were strategies to deal with confounding factors stated?	Yes	Multivariable models and mediation analyses were used to account for relevant covariates.
7	Was appropriate statistical analysis used?	Yes	Regression, mediation, and multiple-testing procedures were appropriate to the study aims.
8	Were the study subjects and the setting described in detail?	Yes	The population-based sampling context, age range, sex

No.	JBI appraisal criterion	Judgement	Scientific justification
			distribution, and measurement methods were described in detail.

Overall methodological appraisal: Generally robust cross-sectional analysis within a cohort; causal direction cannot be established from the contemporaneous measurements.

6. de Souza Marques et al. (2023)

Effects of 12 Weeks of Family and Individual Multi-Disciplinary Intervention in Overweight and Obese Adolescents under Cardiometabolic Risk Parameters: A Clinical Trial

Study design: Randomized controlled trial **Appraisal tool:** JBI Critical Appraisal Tool for Randomized Controlled Trials

No.	JBI appraisal criterion	Judgement	Scientific justification
1	Was true randomization used for assignment of participants to treatment groups?	Yes	Participants were allocated 1:1 using simple randomization generated through an online random-number procedure.
2	Was allocation to treatment groups concealed?	No	The report did not describe allocation concealment before assignment; therefore, selection bias at allocation cannot be excluded.
3	Were treatment groups similar at baseline?	Yes	The intervention groups were broadly comparable at baseline on the reported clinical and anthropometric characteristics.
4	Were participants blind to treatment assignment?	No	Participants necessarily knew whether a parent/guardian attended the intervention; participant blinding was not feasible.
5	Were those delivering treatment blind to treatment assignment?	No	Professionals delivering the family versus individual interventions could not be blinded to group assignment.
6	Were outcome assessors blind to treatment assignment?	Unclear	Laboratory personnel were reported as shielded from the intervention model, but blinding was not clearly established for all outcome assessors and all outcomes.
7	Were treatment groups treated identically other than the intervention of interest?	Yes	Both groups received the same multidisciplinary content and schedule; the intended difference was family participation.

No.	JBI appraisal criterion	Judgement	Scientific justification
8	Was follow-up complete and, if not, were differences between groups in follow-up adequately described and analyzed?	No	A substantial number of randomized participants were not included in the final analysis, and the handling of attrition did not fully protect against attrition bias.
9	Were participants analyzed in the groups to which they were randomized?	No	The final analysis was based on participants who completed the protocol rather than a clearly reported intention-to-treat analysis of all randomized participants.
10	Were outcomes measured in the same way for treatment groups?	Yes	The same clinical, anthropometric, body-composition, and biochemical outcomes were assessed in both groups.
11	Were outcomes measured in a reliable way?	Yes	Biochemical testing and body-composition procedures were standardized; laboratory analyses were performed using specified equipment and procedures.
12	Was appropriate statistical analysis used?	Yes	Repeated-measures and group-comparison analyses were appropriate to the pre/post parallel-group design.
13	Was the trial design appropriate, and were deviations from the standard RCT design accounted for?	Yes	A standard parallel two-arm randomized design was used and was appropriate to the stated comparison.

Overall methodological appraisal: Methodological concerns are driven primarily by absent allocation concealment, unavoidable lack of participant/provider blinding, and incomplete per-protocol follow-up.

7. Martos-Moreno et al. (2020)

Ethnicity Strongly Influences Body Fat Distribution Determining Serum Adipokine Profile and Metabolic Derangement in Childhood Obesity

Study design: Analytical cross-sectional study **Appraisal tool:** JBI Critical Appraisal Tool for Analytical Cross-Sectional Studies

No.	JBI appraisal criterion	Judgement	Scientific justification
1	Were the criteria for inclusion in the sample clearly defined?	Yes	The sample of children and adolescents with obesity and the ethnicity, sex, and pubertal strata were clearly defined.
2	Were objective, standard criteria used for measurement of the condition?	Yes	Obesity and pubertal status were classified using standardized pediatric and clinical criteria.
3	Was the exposure measured in a valid and reliable way?	Yes	Body fat distribution was assessed by whole-body DXA and abdominal MRI with standardized methodology.
4	Were the outcomes measured in a valid and reliable way?	Yes	Metabolic, adipokine, liver-steatosis, glycemic, and lipid outcomes were measured with objective laboratory and imaging methods.
5	Were confounding factors identified?	Yes	Ethnicity, sex, puberty, body-fat distribution, and related metabolic determinants were explicitly considered.
6	Were strategies to deal with confounding factors stated?	Yes	Three-way ANOVA and multivariable logistic-regression analyses were used to account for interacting and independent factors.
7	Was appropriate statistical analysis used?	Yes	The factorial and multivariable statistical approach was appropriate for the stated hypotheses.
8	Were the study subjects and the setting described in detail?	Yes	The clinical population, ethnicity composition, sex balance, pubertal

No.	JBI appraisal criterion	Judgement	Scientific justification
			status, and measurement setting were reported in detail.

Overall methodological appraisal: Generally robust analytical cross-sectional study; residual confounding and cross-sectional temporality remain relevant.

8. van der Heijden et al. (2018)

Obesity-Related Metabolic Risk in Sedentary Hispanic Adolescent Girls with Normal BMI

Study design: Analytical cross-sectional study **Appraisal tool:** JBI Critical Appraisal Tool for Analytical Cross-Sectional Studies

No.	JBI appraisal criterion	Judgement	Scientific justification
1	Were the criteria for inclusion in the sample clearly defined?	Yes	The sample was restricted to post-pubertal sedentary Hispanic adolescent girls with normal BMI, with body-fat subgroups defined explicitly.
2	Were objective, standard criteria used for measurement of the condition?	Yes	BMI and body-fat categories were defined using prespecified objective cut-offs and DXA-derived body composition.
3	Was the exposure measured in a valid and reliable way?	Yes	Regional abdominal and hepatic fat were quantified with MRI/MRS and whole-body fat with DXA.
4	Were the outcomes measured in a valid and reliable way?	Yes	Glucose-insulin indices, inflammatory biomarkers, adipokines, and metabolic outcomes were obtained using objective laboratory methods.
5	Were confounding factors identified?	No	Important potential confounders such as diet history, tobacco exposure, and other lifestyle factors were not comprehensively incorporated into the primary analytical plan.
6	Were strategies to deal with confounding factors stated?	No	Analyses relied primarily on unadjusted correlations and two-group t-tests, without multivariable adjustment for potential confounding.
7	Was appropriate statistical analysis used?	Yes	The statistical tests were conventional and appropriate for simple group comparisons,

No.	JBI appraisal criterion	Judgement	Scientific justification
			although the very small sample limits precision.
8	Were the study subjects and the setting described in detail?	Yes	Participant characteristics, ethnic group, pubertal status, sedentary phenotype, and study procedures were described clearly.

Overall methodological appraisal: Substantial methodological concerns arise from the very small sample and lack of multivariable control for confounding.

9. Duran et al. (2020)

Association of Trunk/Leg Fat Mass Ratio with Low-Density Lipoproteins-Cholesterol and Triglycerides Concentration in Children and Adolescents: A Cross-Sectional, Retrospective Study

Study design: Analytical cross-sectional study **Appraisal tool:** JBI Critical Appraisal Tool for Analytical Cross-Sectional Studies

No.	JBI appraisal criterion	Judgement	Scientific justification
1	Were the criteria for inclusion in the sample clearly defined?	Yes	The NHANES analytical sample was defined by age and availability of DXA and lipid measurements.
2	Were objective, standard criteria used for measurement of the condition?	N/A	Participants were drawn from a population survey rather than a disease-defined cohort; this criterion was not applicable.
3	Was the exposure measured in a valid and reliable way?	Yes	Regional fat mass and trunk/leg fat-mass ratio were derived from standardized NHANES DXA measurements.
4	Were the outcomes measured in a valid and reliable way?	Yes	LDL cholesterol and triglycerides were measured under standardized NHANES laboratory protocols.
5	Were confounding factors identified?	Yes	The analysis identified key confounders including overall fat mass, age, sex, and ethnicity.
6	Were strategies to deal with confounding factors stated?	Yes	Multivariable logistic-regression models adjusted associations for fat-mass index and relevant demographic variables.
7	Was appropriate statistical analysis used?	Yes	Survey-weighted/reference-centile methods and multivariable association analyses were appropriate to the study objectives.
8	Were the study subjects and the setting described in detail?	Yes	NHANES sampling, age range, sample size, sex distribution, and measurement procedures were clearly described.

Overall methodological appraisal: Generally acceptable methodological quality; interpretation is limited by the retrospective cross-sectional design and by trunk/leg ratio being a surrogate rather than direct VAT measurement.

10. Hatch-Stein et al. (2016)

Sex differences in the associations of visceral adiposity, HOMA-IR, and BMI with lipoprotein subclass analysis in obese adolescents

Study design: Analytical cross-sectional study **Apraisal tool:** JBI Critical Appraisal Tool for Analytical Cross-Sectional Studies

No.	JBI appraisal criterion	Judgement	Scientific justification
1	Were the criteria for inclusion in the sample clearly defined?	Yes	Obese adolescents aged 12–18 years were clearly defined using BMI percentile criteria.
2	Were objective, standard criteria used for measurement of the condition?	Yes	Obesity classification was based on standard pediatric BMI thresholds.
3	Was the exposure measured in a valid and reliable way?	Yes	VAT area was measured by DXA using a standardized protocol; HOMA-IR and BMI were calculated using established methods.
4	Were the outcomes measured in a valid and reliable way?	Yes	Fasting lipids and lipoprotein subclasses were measured with standardized laboratory methods.
5	Were confounding factors identified?	Yes	Relevant confounders and effect modification by sex were explicitly considered.
6	Were strategies to deal with confounding factors stated?	Yes	Linear regression and sex-stratified analyses adjusted for relevant covariates.
7	Was appropriate statistical analysis used?	Yes	The regression strategy was appropriate for continuous lipoprotein outcomes and comparisons of VAT, HOMA-IR, and BMI.
8	Were the study subjects and the setting described in detail?	Yes	Age, sex, racial composition, obesity status, clinical setting, and laboratory methods were described adequately.

Overall methodological appraisal: Generally robust analytical cross-sectional study, although generalizability is limited by the predominantly African-American sample.

11. Eloi et al. (2017)

Quantification of Abdominal Fat in Obese and Healthy Adolescents Using 3 Tesla Magnetic Resonance Imaging and Free Software for Image Analysis

Study design: Analytical cross-sectional study **Appraisal tool:** JBI Critical Appraisal Tool for Analytical Cross-Sectional Studies

No.	JBI appraisal criterion	Judgement	Scientific justification
1	Were the criteria for inclusion in the sample clearly defined?	Yes	The prospective observational sample comprised clearly described overweight/obese and healthy adolescents.
2	Were objective, standard criteria used for measurement of the condition?	Yes	Weight-status groups were established using prespecified anthropometric criteria.
3	Was the exposure measured in a valid and reliable way?	Yes	Visceral and subcutaneous abdominal fat were directly measured with 3T MRI using a specified image-analysis approach.
4	Were the outcomes measured in a valid and reliable way?	Yes	Anthropometric, lipid, glucose, and insulin-resistance outcomes were measured using standard clinical and laboratory procedures.
5	Were confounding factors identified?	Unclear	Potential confounding by age, sex, puberty, total adiposity, and other metabolic factors was not comprehensively specified as an a priori confounding structure.
6	Were strategies to deal with confounding factors stated?	No	The principal analyses were correlations and group comparisons without a multivariable strategy to control confounding.
7	Was appropriate statistical analysis used?	Yes	The statistical tests were appropriate for exploratory correlations and two-group comparisons, although power was limited by the small sample.

No.	JBI appraisal criterion	Judgement	Scientific justification
8	Were the study subjects and the setting described in detail?	Yes	Participant groups, mean age, imaging setting, and measurement procedures were clearly reported.

Overall methodological appraisal: Acceptable exploratory study with important concerns regarding small sample size and limited control of confounding.

12. Grunnet et al. (2017)

Adiposity, Dysmetabolic Traits, and Earlier Onset of Female Puberty in Adolescent Offspring of Women With Gestational Diabetes Mellitus: A Clinical Study Within the Danish National Birth Cohort

Study design: Cohort study **Appraisal tool:** JBI Critical Appraisal Tool for Cohort Studies

No.	JBI appraisal criterion	Judgement	Scientific justification
1	Were the exposed and comparison groups similar and recruited from the same population?	Yes	Offspring exposed to maternal GDM and control offspring were recruited within the Danish National Birth Cohort and were drawn from the same underlying source population.
2	Were exposures measured similarly to assign participants to exposed and unexposed/comparison groups?	Unclear	Exposure ascertainment was not fully uniform because GDM identification incorporated self-report and clinical information, and diagnostic criteria varied across some departments.
3	Was the exposure measured in a valid and reliable way?	Unclear	Maternal GDM status was clinically informed but was not verified with one uniform diagnostic standard for every pregnancy; some misclassification is therefore possible.
4	Were confounding factors identified?	Yes	Maternal adiposity, offspring age/sex, offspring BMI, and other relevant determinants were recognized as potential confounders.
5	Were strategies to deal with confounding factors stated?	Yes	Linear and logistic regression models included adjustment and sensitivity analyses for relevant maternal and offspring factors.
6	Were participants free of the outcome at the start of the study (or at the moment of exposure)?	Yes	The adolescent cardiometabolic and pubertal outcomes occurred after the prenatal exposure to maternal GDM.

No.	JBI appraisal criterion	Judgement	Scientific justification
7	Were the outcomes measured in a valid and reliable way?	Yes	Anthropometry, blood pressure, fasting metabolic measures, and DXA outcomes were obtained using standardized clinical procedures.
8	Was the follow-up time reported and sufficient for the outcomes to occur?	Yes	The approximately decade-long interval from pregnancy exposure to assessment at age 9–16 years was sufficient for the outcomes of interest to develop.
9	Was follow-up complete, and if not, were reasons for loss to follow-up described and explored?	Unclear	The analyzed offspring represented a subset of the original birth cohort, and complete participation across the source cohort was not demonstrated.
10	Were strategies to address incomplete follow-up utilized?	Unclear	Sensitivity analyses were reported, but specific attrition-correction methods for differential loss to follow-up were not clearly established.
11	Was appropriate statistical analysis used?	Yes	The regression and sensitivity-analysis framework was appropriate for the exposure-outcome comparisons.

Overall methodological appraisal: Moderate methodological concerns, principally related to exposure ascertainment and potential selection from long-term cohort attrition.

13. Hübers et al. (2017)

Association between individual fat depots and cardio-metabolic traits in normal- and overweight children, adolescents and adults

Study design: Analytical cross-sectional study **Appraisal tool:** JBI Critical Appraisal Tool for Analytical Cross-Sectional Studies

No.	JBI appraisal criterion	Judgement	Scientific justification
1	Were the criteria for inclusion in the sample clearly defined?	Unclear	Data were pooled from the Reference Center for Body Composition, but a single detailed set of eligibility criteria for the pooled sample was not fully transparent.
2	Were objective, standard criteria used for measurement of the condition?	Yes	Weight status and developmental age groups were classified using objective anthropometric and age-based criteria.
3	Was the exposure measured in a valid and reliable way?	Yes	Regional SAT and VAT were measured by whole-body MRI, a reference method for regional adipose tissue.
4	Were the outcomes measured in a valid and reliable way?	Yes	Blood pressure, fasting glucose, insulin, triglycerides, HDL cholesterol, and HOMA-IR were measured using standard procedures.
5	Were confounding factors identified?	Yes	Age, sex, BMI, fat mass, and developmental stage were considered as important confounding or modifying factors.
6	Were strategies to deal with confounding factors stated?	Yes	Partial correlations and multivariable linear regression were used to account for relevant covariates.
7	Was appropriate statistical analysis used?	Yes	The statistical approach was appropriate to age-stratified and multivariable association analyses.
8	Were the study subjects and the setting described in detail?	Yes	Sample sizes, age groups, body-composition methods, and study

No.	JBI appraisal criterion	Judgement	Scientific justification
			setting were reported in sufficient detail.

Overall methodological appraisal: Acceptable methodological quality; pooled cross-sectional data limit causal inference and introduce some uncertainty regarding sample selection.

14. Ishii et al. (2016)

CCL2 level is elevated with metabolic syndrome and CXCL10 level is correlated with visceral fat area in obese children

Study design: Analytical cross-sectional study **Appraisal tool:** JBI Critical Appraisal Tool for Analytical Cross-Sectional Studies

No.	JBI appraisal criterion	Judgement	Scientific justification
1	Were the criteria for inclusion in the sample clearly defined?	Yes	Obese schoolchildren were clearly defined, with age range and degree of overweight described.
2	Were objective, standard criteria used for measurement of the condition?	Yes	Obesity and metabolic syndrome were classified using stated pediatric criteria.
3	Was the exposure measured in a valid and reliable way?	Yes	Visceral fat area was measured by computed tomography using an objective imaging method.
4	Were the outcomes measured in a valid and reliable way?	Yes	Chemokine concentrations and metabolic variables were obtained from fasting blood samples using specified laboratory methods.
5	Were confounding factors identified?	No	The authors acknowledged sex and pubertal-stage heterogeneity as possible sources of variation, but these were not comprehensively incorporated as confounders in the primary analysis.
6	Were strategies to deal with confounding factors stated?	No	No multivariable strategy was reported to adjust the principal VAT-chemokine and metabolic-syndrome associations for major confounding.
7	Was appropriate statistical analysis used?	Yes	Group comparison and correlation analyses were suitable for exploratory objectives, although the sample was small.
8	Were the study subjects and the setting described in detail?	Yes	The clinical population, age distribution, sex composition, obesity definition, and

No.	JBI appraisal criterion	Judgement	Scientific justification
			measurement methods were clearly described.

Overall methodological appraisal: Substantial concerns relate to small sample size, absence of lean controls, and lack of multivariable confounder adjustment.

15. Campos et al. (2019)

Effects of magnitude of visceral adipose tissue reduction: Impact on insulin resistance, hyperleptinemia and cardiometabolic risk in adolescents with obesity after long-term weight-loss therapy

Study design: Quasi-experimental pre-post study **Appraisal tool:** JBI Critical Appraisal Tool for Quasi-Experimental Studies

No.	JBI appraisal criterion	Judgement	Scientific justification
1	Is it clear in the study what is the cause and what is the effect?	Yes	The intervention preceded the post-treatment changes in VAT and cardiometabolic outcomes, establishing clear temporal ordering.
2	Was there a control group?	No	There was no untreated or alternative-treatment control group.
3	Were participants included in any comparisons similar?	Yes	Post-treatment comparison groups were defined by tertiles of VAT reduction and were reported as broadly comparable at baseline.
4	Were participants included in any comparisons receiving similar treatment/care other than the exposure or intervention of interest?	Yes	All participants received the same one-year interdisciplinary lifestyle treatment program before stratification by VAT response.
5	Were there multiple measurements of the outcome both before and after the intervention/exposure?	No	The study reported baseline and post-intervention measurements, but did not include multiple repeated measurements both before and after treatment.
6	Was follow-up complete and, if not, were differences in follow-up adequately described and analyzed?	Yes	The analyzed sample was reported across all three VAT-reduction tertiles, without evidence of differential follow-up affecting the reported comparisons.
7	Were outcomes of participants included in comparisons measured in the same way?	Yes	The same anthropometric, imaging, biochemical, and metabolic procedures were applied across the comparison groups.

No.	JBI appraisal criterion	Judgement	Scientific justification
8	Were outcomes measured in a reliable way?	Yes	VAT and SAT were assessed using standardized ultrasonographic procedures and metabolic outcomes using standard laboratory methods.
9	Was appropriate statistical analysis used?	Yes	Repeated-measures analyses, between-tertile comparisons, correlations, and regression for independent predictors were appropriate.

Overall methodological appraisal: Moderate methodological concerns because the study lacked a concurrent control group and relied on only one pre- and one post-intervention assessment.

16. Fearnbach et al. (2020)

A Pilot Study of Cardiorespiratory Fitness, Adiposity, and Cardiometabolic Health in Youth With Overweight and Obesity

Study design: Analytical cross-sectional study **Appraisal tool:** JBI Critical Appraisal Tool for Analytical Cross-Sectional Studies

No.	JBI appraisal criterion	Judgement	Scientific justification
1	Were the criteria for inclusion in the sample clearly defined?	Yes	Eligibility for youth with overweight to severe obesity and the graded-exercise substudy was described.
2	Were objective, standard criteria used for measurement of the condition?	Yes	Weight status was defined using standard pediatric BMI criteria.
3	Was the exposure measured in a valid and reliable way?	Yes	Abdominal VAT/SAT were quantified by MRI and body composition by DXA using objective methods.
4	Were the outcomes measured in a valid and reliable way?	Yes	VO2peak, fasting laboratory markers, vital signs, and composite cardiometabolic risk measures were assessed using standardized procedures.
5	Were confounding factors identified?	Yes	Physical activity, sedentary time, sex, and other relevant covariates were identified.
6	Were strategies to deal with confounding factors stated?	Yes	Covariate-adjusted analyses were used where appropriate.
7	Was appropriate statistical analysis used?	Yes	Correlation and regression procedures were appropriate for this preliminary exploratory analysis.
8	Were the study subjects and the setting described in detail?	Yes	The small diverse sample, age, sex, race, obesity range, and clinical research setting were described clearly.

Overall methodological appraisal: Acceptable pilot evidence, but precision and generalizability are limited by the very small analytic sample.

17. Bland et al. (2022)

Visceral adipose tissue and cardiometabolic risk factors in young Hispanic and non-Hispanic girls

Study design: Analytical cross-sectional study **Appraisal tool:** JBI Critical Appraisal Tool for Analytical Cross-Sectional Studies

No.	JBI appraisal criterion	Judgement	Scientific justification
1	Were the criteria for inclusion in the sample clearly defined?	Yes	The adolescent-girl sample and eligibility procedures were clearly described.
2	Were objective, standard criteria used for measurement of the condition?	N/A	The cohort was not selected on the basis of a single disease diagnosis; this criterion was not applicable.
3	Was the exposure measured in a valid and reliable way?	Yes	VAT was derived from DXA using a standardized protocol.
4	Were the outcomes measured in a valid and reliable way?	Yes	Triglycerides, cholesterol, HOMA-IR, C-reactive protein, blood pressure, and related outcomes were assessed using established clinical methods.
5	Were confounding factors identified?	Yes	Age, ethnicity, Tanner stage, and BMI percentile were specified as important covariates and effect modifiers.
6	Were strategies to deal with confounding factors stated?	Yes	Multiple linear regression models adjusted for the identified covariates and formally evaluated interactions with BMI category.
7	Was appropriate statistical analysis used?	Yes	The regression and stratified analyses were appropriate to the study question.
8	Were the study subjects and the setting described in detail?	Yes	The sample size, ethnic composition, age group, BMI categories, and measurement setting were adequately reported.

Overall methodological appraisal: Generally robust analytical cross-sectional study, with residual confounding and limited causal inference inherent to the design.

18. Wang et al. (2025)

Exploring Visceral Fat as a Screening Marker for Cardiometabolic Risk in Children and Adolescents

Study design: Cross-sectional derivation study with longitudinal cohort validation **Appraisal tool:** JBI Critical Appraisal Tool for Cohort Studies (applied to the longitudinal validation component, while considering the cross-sectional derivation phase)

No.	JBI appraisal criterion	Judgement	Scientific justification
1	Were the exposed and comparison groups similar and recruited from the same population?	Yes	The derivation and validation samples were population-based pediatric cohorts with clearly defined eligibility and follow-up procedures; risk groups were derived within the same cohort structures.
2	Were exposures measured similarly to assign participants to exposed and unexposed/comparison groups?	Yes	VFA was measured using the same standardized body-composition approach within each cohort for exposure classification.
3	Was the exposure measured in a valid and reliable way?	Yes	DXA-derived visceral fat area was measured objectively using standardized protocols, although the authors acknowledged modest bias compared with MRI/CT.
4	Were confounding factors identified?	Yes	Age, sex, anthropometry, and relevant cardiometabolic determinants were considered in cutoff derivation and predictive analyses.
5	Were strategies to deal with confounding factors stated?	Yes	Sex- and age-specific thresholds, regression models, and stratified analyses were used to account for key confounding and effect modification.
6	Were participants free of the outcome at the start of the study (or at the moment of exposure)?	Unclear	The report did not clearly establish that all participants were free of every cardiometabolic outcome at baseline before prediction of two-

No.	JBI appraisal criterion	Judgement	Scientific justification
			year risk; this criterion was therefore judged unclear.
7	Were the outcomes measured in a valid and reliable way?	Yes	Hypertension, hyperglycemia, dyslipidemia, and clustered cardiometabolic risk were assessed using prespecified objective clinical criteria.
8	Was the follow-up time reported and sufficient for the outcomes to occur?	Yes	Two-year longitudinal follow-up was adequate for short-term validation of cardiometabolic risk, although insufficient for adult cardiovascular endpoints.
9	Was follow-up complete, and if not, were reasons for loss to follow-up described and explored?	Yes	A large validation cohort completed follow-up assessments and participant flow was reported.
10	Were strategies to address incomplete follow-up utilized?	Yes	Participant selection, exclusion criteria, and analytic handling of the validation sample were described sufficiently to mitigate incomplete follow-up concerns.
11	Was appropriate statistical analysis used?	Yes	ROC analysis, risk estimation, sex-specific cutoffs, and longitudinal validation procedures were appropriate to the screening-marker objective.

Overall methodological appraisal: Generally strong large-sample evidence for short-term risk stratification, with uncertainty regarding baseline outcome-free status and limited representativeness from nonrandom sampling.

19. Yan et al. (2019)

Abdominal visceral and subcutaneous adipose tissues in association with cardiometabolic risk in children and adolescents: the China Child and Adolescent Cardiovascular Health (CCACH) study

Study design: Analytical cross-sectional study **Appraisal tool:** JBI Critical Appraisal Tool for Analytical Cross-Sectional Studies

No.	JBI appraisal criterion	Judgement	Scientific justification
1	Were the criteria for inclusion in the sample clearly defined?	Yes	The CCACH analytical sample was clearly defined by age, urban sampling framework, DXA availability, and cardiometabolic measurements.
2	Were objective, standard criteria used for measurement of the condition?	N/A	The study was population-based rather than disease-selected; this item was not applicable.
3	Was the exposure measured in a valid and reliable way?	Yes	VAT and SAT were quantified by DXA using standardized procedures.
4	Were the outcomes measured in a valid and reliable way?	Yes	Blood pressure, lipid, glucose, and other metabolic risk factors were assessed using objective standardized methods and prespecified thresholds.
5	Were confounding factors identified?	Yes	Region, family income, age, pubertal development, physical activity, smoking, and overall fat mass were identified as relevant covariates.
6	Were strategies to deal with confounding factors stated?	Yes	Multivariable models adjusted for these factors and included additional adjustment for fat-mass index.
7	Was appropriate statistical analysis used?	Yes	The large-sample multivariable and sex-stratified analyses were appropriate to the objectives.
8	Were the study subjects and the setting described in detail?	Yes	The urban Chinese setting, sample size, age range, sex-specific analyses, and measurement

No.	JBI appraisal criterion	Judgement	Scientific justification
			methods were comprehensively described.

Overall methodological appraisal: Generally robust analytical cross-sectional evidence, with the principal limitation being inability to determine temporality or causality.

20. Yasuda et al. (2021)

Adiponectin, ALT and family history as critical markers for the development of type 2 diabetes in obese Japanese children

Study design: Analytical cross-sectional study **Appraisal tool:** JBI Critical Appraisal Tool for Analytical Cross-Sectional Studies

No.	JBI appraisal criterion	Judgement	Scientific justification
1	Were the criteria for inclusion in the sample clearly defined?	Yes	The outpatient sample of obese Japanese children and the T2D, metabolic-syndrome, and comparison groups were clearly described.
2	Were objective, standard criteria used for measurement of the condition?	Yes	Obesity, type 2 diabetes, and metabolic syndrome were classified using stated clinical criteria.
3	Was the exposure measured in a valid and reliable way?	Yes	VAT and SAT were measured from computed-tomography images using objective methods.
4	Were the outcomes measured in a valid and reliable way?	Yes	Adiponectin, leptin, liver enzymes, glucose-related variables, and other metabolic measures were obtained using standard clinical laboratory methods.
5	Were confounding factors identified?	Unclear	Several biologically relevant factors were discussed, but a comprehensive prespecified confounding structure was not clearly presented.
6	Were strategies to deal with confounding factors stated?	No	The primary analyses were largely unadjusted group comparisons and ROC analyses rather than multivariable adjustment for potential confounders.
7	Was appropriate statistical analysis used?	Yes	Group comparisons and ROC analyses were appropriate for marker discrimination, although they do not fully address confounding.

No.	JBI appraisal criterion	Judgement	Scientific justification
8	Were the study subjects and the setting described in detail?	Yes	The clinical setting, age group, obesity status, group sizes, and measurement procedures were adequately described.

Overall methodological appraisal: Acceptable exploratory marker study with important concerns regarding confounding and the cross-sectional comparison of established clinical groups.

Suggested wording for the Methods section

Methodological quality was assessed using design-specific Joanna Briggs Institute (JBI) critical appraisal tools. Analytical cross-sectional studies were evaluated using the JBI tool for analytical cross-sectional studies, cohort studies using the JBI cohort tool, the randomized trial using the JBI randomized controlled trial tool, and the non-randomized intervention study using the JBI quasi-experimental tool. Each item was rated as Yes, No, Unclear, or Not Applicable. Appraisal was performed at the domain level, without applying an arbitrary numerical quality threshold. Methodological limitations were considered when interpreting the consistency and strength of the evidence regarding visceral adipose tissue and cardiometabolic risk stratification.
