# Supplementary material for: Beyond BMI: The Role of Visceral Adipose Tissue in Cardiometabolic Risk Stratification Among Children and Adolescents with Obesity—A Systematic Review
Source: Medicina (Kaunas). 2026 Sep 24;62(10):1851. doi: 10.3390/medicina62101851 (PMC13707073; doi:10.3390/medicina62101851)
Supplement: Supplementary file 1 [file medicina-62-01851-s001.zip › Table S3 Supplementary_Search_Strategy.docx.pdf]

Supplementary File 1. Search Strategy and Study Selection

Beyond BMI: The Role of Visceral Adipose Tissue in Cardiometabolic Risk Stratification Among Children and Adolescents with Obesity — A Systematic Review

Search strategy

A systematic literature search was conducted in PubMed and Scopus for English-language studies published between 1 January 2016 and 28 February 2026. The search strategy combined terms related to visceral adiposity, adolescents and youth, and cardiovascular or cardiometabolic risk. No sex-related search terms or restrictions were applied. Eligibility screening covered the predefined age range of 6–19 years, and the retrieved records included studies involving both children and adolescents. The complete database-specific search strategies are presented below.

**Database**	**Search strategy**
PubMed<br>n=391	("visceral adipose tissue"[MeSH] OR "visceral fat" OR "abdominal fat" OR "intra-abdominal fat")<br>AND<br>(adolescent*[MeSH] OR adolescen* OR teen* OR youth*)<br>AND<br>("cardiovascular risk"[MeSH] OR "cardiovascular disease" OR "cardiometabolic risk" OR hypertension OR dyslipidemia OR insulin resistance OR "metabolic syndrome")
Scopus<br>n=322	("visceral adipose tissue" OR "visceral fat" OR "abdominal fat" OR "intra-abdominal fat")<br>AND<br>(adolescent* OR adolescen* OR teen* OR youth*)<br>AND<br>("cardiovascular risk" OR "cardiovascular disease" OR "cardiometabolic risk" OR hypertension OR dyslipidemia OR insulin resistance OR "metabolic syndrome")<br><br>Limited to: Article<br>Limited to: Medicine<br>Limited to: English

Manual screening of the reference lists of eligible studies did not identify any additional records meeting the predefined inclusion criteria.

Study selection

A total of 713 records were identified through searches of PubMed and Scopus. Before title and abstract screening, 277 duplicate records and 44 records removed for other reasons were excluded, leaving 392 records for screening. Of these, 332 records were excluded because they were irrelevant or outside the scope of the review. Consequently, 60 full-text reports were assessed for eligibility. Following full-text assessment, 40 reports were excluded for the following reasons: VAT was not assessed using an eligible imaging modality (n = 18), cardiometabolic risk markers were not reported or assessed (n = 10), exclusionary comorbidities were present (n = 7), and the study population did not meet the predefined age criteria (n = 5). Ultimately, 20 studies met the predefined eligibility criteria and were included in the systematic review.

Two reviewers independently screened the titles and abstracts, assessed the full-text reports for eligibility, and extracted the relevant study data using a standardized extraction form. Any disagreements concerning study selection or data extraction were resolved through discussion or, when necessary, consultation with a third reviewer.
